# Platelets and Cardioprotection: The Role of Nitric Oxide and Carbon Oxide

**DOI:** 10.3390/ijms24076107

**Published:** 2023-03-24

**Authors:** Isabella Russo, Cristina Barale, Elena Melchionda, Claudia Penna, Pasquale Pagliaro

**Affiliations:** Department of Clinical and Biological Sciences of Turin University, Orbassano, I-10043 Turin, Italy

**Keywords:** platelets, preconditioning, remote conditioning, nitrosating agents, gasotransmitters

## Abstract

Nitric oxide (NO) and carbon monoxide (CO) represent a pair of biologically active gases with an increasingly well-defined range of effects on circulating platelets. These gases interact with platelets and cells in the vessels and heart and exert fundamentally similar biological effects, albeit through different mechanisms and with some peculiarity. Within the cardiovascular system, for example, the gases are predominantly vasodilators and exert antiaggregatory effects, and are protective against damage in myocardial ischemia-reperfusion injury. Indeed, NO is an important vasodilator acting on vascular smooth muscle and is able to inhibit platelet activation. NO reacts with superoxide anion (O_2_(^−^•)) to form peroxynitrite (ONOO(^−^)), a nitrosating agent capable of inducing oxidative/nitrative signaling and stress both at cardiovascular, platelet, and plasma levels. CO reduces platelet reactivity, therefore it is an anticoagulant, but it also has some cardioprotective and procoagulant properties. This review article summarizes current knowledge on the platelets and roles of gas mediators (NO, and CO) in cardioprotection. In particular, we aim to examine the link and interactions between platelets, NO, and CO and cardioprotective pathways.

## 1. Introduction

Among cardiovascular diseases, myocardial infarction remains the leading cause of death and morbidity worldwide, representing 46% of all cardiovascular deaths in men and 38% in women [1]. The major challenge in facing myocardium loss after infarcting ischemia, is to reduce the so-called ischemia reperfusion injury (IRI) to limit ischemia/hypoxia-driven oxidative stress, inflammation, and intracellular Ca^2+^ overload and all forms of cell death [2,3]. IRI is due to a series of events in which several mechanisms play a pivotal role. These events and mechanisms include calcium overload, dissipation of mitochondrial membrane potential, endothelial dysfunction, platelet activation, microembolization, immune activation, autophagy, and, eventually, all forms of cell death [3]. In this regard, several therapeutic approaches to spare the ischemic myocardium have been proposed and different intracellular mechanisms are proven to be involved both in the pre- and post-ischemic phase and can, therefore, be targeted to achieve a more effective protected state of the myocardium. All of these mechanisms in some way involve nitric oxide (NO) and reactive oxygen species (ROS), which also lead to the formation of reactive nitrogen species (RNS) [4,5]. In determining reperfusion injury, the first minutes of reperfusion are really important. In particular, mitochondrial permeability transition pore (MPTP) opening and subsequent cell death by apoptosis and necrosis by rupture of the cell membrane of cardiomyocytes play a central role in this phase. In triggering the long-lasting opening of MPTPs, redox stress, mitochondrial Ca^2+^ overload, acute restoration of a physiological pH, and adenosine triphosphate (ATP) depletion are involved [6], followed by inflammatory response [7]. Molecular oxygen reduction induces ROS production during ischemia, but, especially, during early reperfusion, and it has been proposed that these ROS induce several lesions. In particular, ROS modulate the activity of enzymes, such as cytochrome and xanthine oxidase, cyclooxygenase, and caspases, leading to catecholamine oxidation and polymorphonuclear (PMN) activation [6]. However, it is important to remember that small amounts of ROS in the early stages of reperfusion are protective, as reported by several researchers [8,9,10]. Another important gas involved in platelet function and cardioprotection is carbon monoxide (CO).

The role played by gases and platelets in IRI is double and opposite; in fact, they appear to be involved in IRI [11,12,13] but also in the cardioprotection directly [14,15] or with production of cardioprotective molecule [16,17]. It is known that in the first phase of reperfusion, platelet activation occurs with consequent accumulation in the ischemic area [18]. Platelets participate in IRI by several mechanisms: aggregation and microthrombi formation, platelet–leukocyte aggregation, release of exosomes and vasoconstrictors, plasma membrane-derived vesicles (PMVs) and apoptotic body formation, and spinal afferent nerve activation [19]. In particular, activation and subsequent platelet–leukocyte interactions and platelet aggregation with the formation of microthrombi in small cardiac vessels and capillaries exert a key role in causing cardiac tissue damage [19]. Platelet activation and interactions with leukocytes and vascular endothelium are followed by the release of the platelet granules content and, thus, the development of an inflammatory response [13]. In this scenario the response of cardioprotective maneuvers is fundamental to reduce the IRI. In particular, gasotransmitters are emerging as cardioprotective factors interconnected to platelet function. In light of this, to the best of our knowledge, this review is the first to emphasize and examine the close relationships between gases, such as NO, derivatives and carbon monoxide (CO), and platelet function and interconnections with cardioprotection. These interconnections are also examined based on the results obtained from our original studies, e.g., [8,14].

## 2. Cardioprotection

Myocardial cell death due to IRI is one of the main causes of morbidity and mortality in the western world which can be reduced through cardioprotection protocols. Platelets and gasotransmitters play a role in cardioprotective scenarios. The first studies in the field of cardioprotection were by Maroko and colleagues in the early 1970s, subsequently the group of Murry, Reimer, and Jennings described the phenomenon of ischemic preconditioning (IP) in 1986 [20,21,22]. Another important step toward cardioprotection is the study by Vinten Johansen’s group in which intermittent interruption of coronary blood flow in the very early stage of reperfusion leads to cardioprotection [23]. This protection was referred to as postconditioning (PostC) and consists of short cycles of IR at the end of prolonged ischemia [23]. These cardioprotective protocols are able to significantly reduce the IRI by activating signaling pathways capable of modifying cardiac function with a reduction of infarct size and mechanical dysfunction.

All these protective protocols start from the release of ligands that induce the activation of the molecular pathway in the ischemic myocardium. This ligand–receptor interaction activates complex cascades involving several molecules, e.g., membrane G protein, growth factor receptors, signaling enzymes, such as NOS/cGMP-dependent protein kinase G (PKG), protein kinase C (PKC), ATP-sensing potassium channels (KATP), and ROS [24,25]. The end effector is the MPTP in which the protective cascade prevents pore formation, leading to protection [26,27]. The first pathway involved in cardioprotection has been referred to as reperfusion injury salvage kinase (RISK) [28].

In 2009, Lecour et al. reported another important protective signaling pathway, enhancement of survival activating factor (SAFE) [29]. Other kinases have been activated in this pathway compared to RISK, especially the Janus kinase (JAK)/signal transducer and activator of transcription 3 (STAT3) pathway, which inhibits MPTP opening, thereby promoting cardiac survival [29,30]. Another important and fundamental pathway involved in cardioprotection, with relation to platelets, is represented by the NO/PKG pathway [31]. This pathway is activated by NO and natriuretic peptides (NPs; e.g., ANP (atrial NP), BNP (brain NP), and CNP (C-type NP)); in particular, NO can start this pathway by activating the soluble guanylate cyclase (sGC), while NPs activate the particulate GC (pGC). Both sGC and pGC, when activated, produce cGMP as a second messenger. Undoubtedly, cGMP exerts its physiological actions largely through targeting PKG. However, the effects brought about by cGMP can be significantly different depending on its subcellular localization [31,32]. PKG induces activation of a protein on the mitochondrial outer membrane (MOM), with consequentially opening of the mitochondrial KATP channel (mitoKATP) on the mitochondrial inner membrane, thus increasing production of mitochondrial ROS. Also in this pathway, the end effector is represented by the inhibition of MPTP and reduction in cell death [31,32].

Another important cardioprotective protocol is represented by remote ischemic conditioning (RIC), in which a non-target organ or tissue is exposed to short periods of IR for conditioning with a significative reduction of cardiac infarct size [33]. This protocol induces protection if applied before (remote ischemic preconditioning, RIPC), during (remote ischemic perconditioning, RiPerC), or after (remote ischemic postconditioning, RiPostC) the myocardial ischemic insult.

Several studies reported that the signal transfer of RIC was dependent by the humoral and neuronal pathways. In particular, preclinical experiments report the possibility of achieving humoral signal transfer, in which the cardioprotection of RIC is transferred with plasma or plasma-dialysate from conditioned donors to the heart isolated from a rodent, subjected to ex vivo ischemia/reperfusion (IR). It appears that RIC attenuates platelet activation in patients with coronary artery disease after treadmill exercise [34] or after coronary angiography [35]. Although RIC attenuates platelet activation, it is unclear whether or not RIC-induced cardioprotective signal transfer involves platelets or its derivatives, such as exosomes and PMVs [15,36].

## 3. Some Aspects of Platelet Activation

Platelets are small anucleated cells derived from the fragmentation of megakaryocytes and represent a key component involved in the intricate process of hemostasis together with vascular endothelium, coagulation, and fibrinolysis. Platelets contain three types of granules (i.e., alpha, dense, and lysosomes) and the secretion of molecules stored in these granules in response to stimulus is able to modulate aggregation and thrombus formation [37]. Platelets may be activated by various compounds, including collagen, thromboxane A_2_ (TXA_2_), coagulation factors (thrombin), adenosine diphosphate (ADP), and serotonin, by their binding to receptors on the platelet surface. Alterations of platelet function can result in pathological consequences, such as arterial thrombosis or hemorrhage. Platelets are also known for their ability to influence immune response, tumor progression, and inflammation [38]. Specifically, during sepsis, platelet hyperactivation can exacerbate coagulation and inflammation by promoting endothelial dysfunction, neutrophil extracellular traps (NETs) formation, and generation of microthrombi [39,40]. Platelet responses include adhesion to adhesive molecules such as collagen, secretion of compounds from their granules leading to form a hemostatic plug or thrombus [40].

The process by which platelets form a plug is known as primary hemostasis, whereas secondary steps activate the procoagulant system. Platelet events involved in primary hemostasis mainly consist in adhesion, secretion, and aggregation [41]. When platelets are stimulated, hydrolysis of phosphoinositide and synthesis of eicosanoids represent two key interrelated signal transduction cascades leading to platelet activation, which will result in the elevation of intracellular calcium levels. When intraplatelet Ca^2+^ levels exceed a specific threshold, platelets undergo rapid shape change driven by the actin cytoskeleton and shift from the resting discoid to a flattened morphology with the extension of multiple filopodia and lamellipodia [42,43].

### NO and Platelets

**Figure 1:**
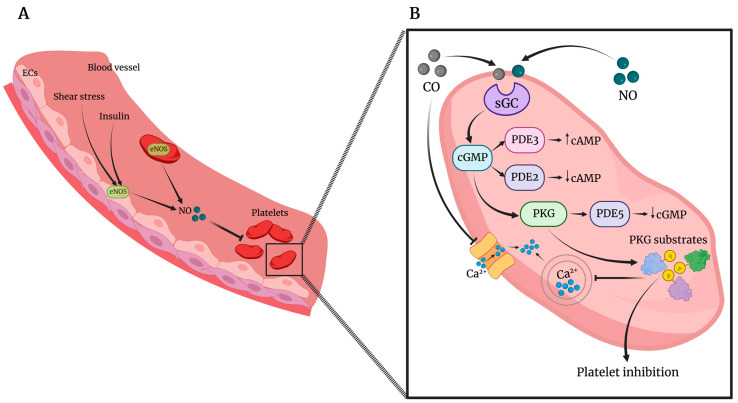
(**A**). Nitric oxide (NO) in blood vessels is mainly synthetized by endothelial nitric oxide synthase (eNOS), expressed by endothelial cells (ECs), and it is enhanced by stimuli such as insulin and shear stress. Platelets could also express eNOS, representing, therefore, another source of NO in the blood stream. (**B**). In platelets, NO enhances guanosine 3’,5’-cyclic monophosphate (cGMP) synthesis through the activation of soluble guanylate cyclase (sGC). cGMP activates phosphodiesterase 3 (PDE3) and PDE2 activity, modulating platelet cyclic adenosine monophosphate (cAMP) levels. cGMP activates PKG, leading to the activation of PDE5 and reduction of cGMP levels, and is responsible for the phosphorylation of many substrates involved in mechanisms of inhibition of platelet activity, including intracellular Ca^2+^ release. Similarly, CO also stimulates the activation of sGC and inhibits Ca^2+^ entry.

Platelet function is regulated by a dynamic equilibrium between agonists and inhibitory substances, including the well-documented gasotransmitter NO. NO inhibits platelets by preventing platelet adhesion, activation, aggregation, and disaggregating previously aggregated platelets [44]. The NO/cGMP/PKG pathway has been postulated as the main mechanism by which NO inhibits platelet function in vivo and in vitro [45] (Figure 1). NO acting on platelets mainly derives from endothelial cells (which represent the principal source of vascular NO) stimulated by different stimuli, including shear stress, vascular endothelial growth factor (VEGF), insulin, bradykinin, and other stimuli able to increase intracellular calcium concentration and activate eNOS. However, a large body of evidence reported platelets as sources of the two NOS isoforms, i.e., eNOS and inducible NOS (iNOS), albeit there is not unanimous agreement on both expression and enzymatic activity of NOS in platelets [46,47]. In other words, platelet ability to produce itself NO that acts to prevent also other circulating platelets has not been definitely clarified yet. Interestingly, it has been demonstrated that human platelets can be divided into two distinct populations, positive or negative, for the expression of functionally active eNOS [48], thus reflecting the heterogeneity of platelet population and megakaryocytes. Indeed, the questions about the potential NOS expression in distinct platelet populations and whether platelets themselves are able to produce NO remain still open and new experimental approaches are needed to solve them.

NO regulates cGMP levels and the cGMP inhibitory effects predominantly depend on the PKG, the major effector of cGMP signaling in the cardiovascular system [49,50,51,52,53] (Figure 1).

Specifically, PKG interferes with platelet function by inhibiting many agonist-induced activation pathways, including the reorganization of cytoskeleton, secretion of platelet granules [45,54], the increase in intraplatelet calcium levels [55], and integrin activation [56]. Of note, besides the activation of sGC, NO as well as NO metabolites can influence platelet response by cGMP-independent mechanisms even if at higher concentrations than those needed for sGC activation [57,58]. Mechanisms of NOS activation in platelets as well as in other cells include increase in intracellular calcium levels, phosphorylation at Ser^633^, Ser^1177^, and Thr^495^, and the interaction with proteins such as caveolin, Hsp70, and Hsp90 [59,60].

Activation of platelet eNOS has been shown to be promoted also by β_2_-adrenoceptor [61], insulin [62], and acetylsalicylic acid [63] by mechanisms independent of calcium and dependent on NOS phosphorylation. Platelet NOS activity is increased by cAMP/PKA pathway activated by adenosine, forskolin, and, potentially, by every antiaggregating substance able to increase intraplatelet cAMP via receptor-dependent and -independent mechanisms [64] (Table 1).

Other studies showed calcium-dependent platelet eNOS stimulation [65,66], as well as eNOS activation by Thr^495^ de-phosphorylation [67]. As mentioned, NO is an important negative regulator of signal transmission during blood platelet activation [68,69], then reduced NO synthesis or action are implicated in platelet hyperreactivity [70].

As mentioned, physiological hemostasis is the result of a dynamic state between pro- and anticoagulation processes, which can be influenced by gas mediators including NO, then any imbalance between the pro- and anti-coagulant processes may be responsible for bleeding or blood clots. An important cause of reduced protective action of NO on platelets is due to increased levels of ROS. Indeed, ROS represent a well-established second messenger for intraplatelet signal and both increased ROS synthesis and impaired ROS neutralization in platelets are deeply involved in the thrombotic process [71,72]. In particular, the rapid reaction of NO with superoxide anion (O_2_^−^) leads to peroxynitrite (ONOO^−^) generation [41,73], an oxidant agent able to activate or inhibit the hemostatic functions of platelets [74,75]. Targets of ONOO^−^ action in platelets are lipids and proteins, resulting in nitration of tyrosine and carbonylation of many proteins [76,77,78] and lipid peroxidation that causes changes in the structure of platelet membrane and alterations of membrane receptors. In light of this, platelet redox imbalance found in some metabolic diseases, such as diabetes, dyslipidemia, and metabolic syndrome, may promote a prothrombotic state leading to occlusive arterial thrombi associated with myocardial infarction and stroke [72,79,80,81,82,83].

Given that insulin has been shown to inhibit platelet function also through a rapid increase in NO-mediated cGMP and cAMP [84] and platelets are targets of insulin action, in conditions of insulin-resistance such as central obesity, type 2 diabetes, hypertension, the inhibitory actions of insulin on platelets are impaired [85]. Specifically, platelets from insulin-resistant subjects show multi-step defects of NO/cGMP/PKG [86]. Since a crucial feature to initiate signaling events leading to platelet activation is the increase in intracellular Ca^2+^ [87] and cGMP exerts its effects on platelets mainly through a reduction of intracellular Ca^2+^ [54], these findings indicate the presence in insulin-resistance of alterations in Ca^2+^ fluxes handling. However, lifestyle interventions aimed at reducing body weight can restore platelet sensitivity to NO/cGMP/PKG accompanied by an improvement of insulin resistance and a decrease in inflammation [88,89]. Platelets isolated from obese subjects have been shown to exhibit reduced NO-dependent antiplatelet activity, both of endogenous and NO donor origin. The reason for this seems to be due to the lower insulin sensitivity typical of these subjects. From a molecular point of view, this suggests lower GC activation. In these conditions, NO deficiency is compensated by the use of antioxidant substances such as N-acetyl-L-cysteine (NAC) or antioxidant enzymes such as superoxide dismutase (SOD), which inactivates the extracellular superoxide anion [90]. Recent studies have shown that both NO donors and cGMP analogs are able to increase the antiplatelet activity of drugs ligating the P2Y12 receptor [53].

On the other hand, platelet NO production has also been recognized as a novel mechanism of platelet activation. As known, the disproportionate host inflammatory response to pathogens during sepsis may be due to the effects of a cytokine storm characterized by a huge production and action of inflammatory cytokines [91,92]. Some of these inflammatory factors may have a role in the activation of the inducible isoform of NO-synthase in platelets. For example, platelets from dengue patients show increased iNOS expression and NO production. In this clinical setting, platelet NO production correlates with the inflammatory cytokine IL-1β and severity of disease, data also confirmed by in vitro IL-1β stimulation which reproduced platelet response in vivo [93].

## 4. Role of NO in Cardioprotection and Platelets

The signal transduction pathways involved in cardioprotection include an important contribution both from redox signaling due to the production of ROS but also from NO itself and its derivatives, such as nitroxyl (HNO) [94,95].

Following the production of NO, important modifications of proteins by S-nitrosylation were detected. The role of NO in cardioprotection has been extensively studied. The intervention of platelets in the cardioprotective field is given by the production of molecules able to exert their protective effect by directly or indirectly inducing the production of NO [96,97].

An important molecule of platelet origin, platelet activating factor phosphoglyceride (PAF), is able to act as an autocrine/paracrine mediator on various experimental models, including cardiomyocytes and isolated hearts. While at high concentrations (1–10 nmol/L) it shows direct and indirect negative effects on the heart, at low concentrations (pM), PAF demonstrates a protective effect, similar to ischemic preconditioning [12,16]. The cardioprotective action of PAF is due to the activation of the RISK pathway, particularly with PKC/protein kinase B (Akt)/NOS involvement [16,98]. The cardioprotective action exerted by low doses of PAF also depends on NO-mediated S-nitrosylation of proteins involved in calcium transport, such as L-type Ca^2+^ channels; thus, reducing Ca^2+^ overload during myocardial ischemia–reperfusion [98]. Platelets allow the release of sphingosine-1-phosphate (S1P) produced from membrane sphingosine through the action of sphingosine kinase [17].

The protective action of S1P takes place directly through the S1P1, S1P2, and S1P3 receptors present in cardiomyocytes, with consequent activation of the RISK and enhancement of survival activating factor (SAFE) protective pathways [36]. S1P itself is an important activator of endothelial NO synthase (eNOS) at the platelet level [99]. It is well known that purinergic type 2 receptor subtypes (ADP-binding P2Y1, purinergic Y-type 12 (P2Y12), and ATP-binding P2X1) are involved in both aggregation and platelet shape change. Recently, it has been observed that P2Y12 inhibitors, including prasugrel [100], cangrelor [101], and ticagrelor [102], also display cardioprotective effects [103]. Currently, the potential cardioprotective mechanism of the action of P2Y12 inhibitors is not fully understood. Their cardioprotective action could be due to phosphorylation of sphingosine, activation of PI3k/Akt signaling pathways, and/or blockade of the ENT1 transporter, the adenosine transported, resulting in increased tissue levels of adenosine, thereby reducing cardiac damage, by mechanisms other than those attributed to inhibition of the NLRP3 complex [104,105,106,107]. Recent studies have shown that in patients aged 70 years and older at high risk of bleeding, clopidogrel is a favorable alternative to ticagrelor and prasugrel, as it results in fewer bleeding events without an increase in the combined endpoint of death from all causes, myocardial infarction, stroke, and hemorrhage [103,108].

## 5. The Role of CO in Cardioprotection and Platelet

Differently from NO, only a limited number of studies have been carried out on CO effects on platelets and CO and cardioprotection.

### 5.1. CO and Cardioprotection

The cardioprotective action of CO is mediated by the opening of KATP and consequent inhibition of the opening of the MPTP. It has been observed that CO donors (CORM) [109] demonstrate a protective effect, reporting a certain influence of sex on cardioprotection. In fact, as known from the literature, sex determines the expression of specific genes and proteins involved in protection of mitochondrial and myocardial function, such as Akt [109].

The protective action exerted by CO at low doses allows the maintenance of mitochondrial function, in fact, it has been observed that in these conditions it is able to maintain the mitochondrial membrane potential stable, which is altered in ischemia/reperfusion injury [97].

It has also been reported that CO is able to modulate the synthesis of mitochondrial ROS, the activity of some hemoproteins, including cytochrome c, and inflammation (inflammasome-dependent) [110,111]. Although mitochondrial ROS are involved in cardioprotection [8,9,10,112] and mitochondrial ROS induced by NO are cardioprotective [113], it is not known if ROS generated by exogenous CO are cardioprotective.

### 5.2. CO and Platelets

Although early studies showed stimulating CO effects on platelet aggregation, more recent studies have indicated a CO ability to inhibit platelet aggregation and release of ADP and serotonin from their granules [114,115] (Table 1).

CO and NO share some chemical and biological properties [116]. Indeed, exogenously added CO inhibits platelet aggregation mainly by elevating intracellular levels of cGMP [116]. Gaseous CO shows antiaggregating properties with similarity with NO in increasing sGC activity as result of direct binding of CO to the iron present in the heme moiety of sGC, even if CO is 30–100 times less potent than NO [115,117]. There is agreement on the concept that the inhibitory actions of CO on platelets are relatively low in comparison to those exerted by the endothelium-released agents NO and prostacyclin [118]. Actually, only high concentrations of gaseous CO (100%) seem to reduce platelet aggregation via sGC activation [117].

Platelets are the target but also the source of CO given that platelets express heme oxygenase 1 (HO-1) and are involved in multiple steps of heme and bilirubin metabolism [119]. In the presence of HO-1 activators, such as hemin- and sodium arsenite, platelet agonist-induced aggregations are reduced and margination and rolling are prevented, these effects are abolished by the HO-1 inhibitor zinc protoporphyrin IX (ZnPP-IX) and reproduced by CO [120,121]. Even if under basal condition, HO-1 does not significantly influence platelet-dependent clot formation in vivo, in the presence of increased HO-1 production, platelet-dependent thrombus formation is suppressed [122]. These findings induced the authors to suggest that the enhanced HO-1 expression may be a mechanism able to reduce platelet activation under prothrombotic states. A study using HO-1 knockout mice found normal platelet number, bleeding time, and platelet aggregating characteristics, but accelerated thrombosis, at least partially, due to platelet activation, which was rescued by inhaled CO [123] or CO-donor [124].

The unexpected physiological roles of CO in the cardiovascular system have justified the development of CO-releasing compounds [125] and the evaluation of their effects also on platelet function. Actually, CO-donor compounds have been shown to effectively inhibit human platelets without involving the activation of sGC [114] even if NO- and CO-mediated effects on platelets seem to be interlinked given that the inhibition of sGC increases the inhibitory CO effects.

CO is able to reduce the calcium signal elicited by platelet agonists by a direct effect on calcium entry [126], thus, confirming a role for HO activity in modulating platelet response. Different mechanisms could explain CO effect on intraplatelet calcium levels. On the one hand, CO can induce a cGMP-mediated decrease in calcium release from intracellular stores or an acceleration in the rate of its back-sequestration. On the other hand, CO shows the ability to directly inhibit the pathway involved in calcium entry [126]. The direct role of CO on capacitative calcium entry may be responsible for its antiaggregatory action.

Other cGMP-independent mechanisms by which CO-donors can inhibit platelet function include the CO ability to interfere with glycoprotein-mediated HS1 phosphorylation, a signaling molecule involved downstream of glycoprotein activation. In particular, it has been shown that during lipopolysaccharide (LPS)-induced platelet activation, the signal transmitted between membrane glycoproteins and HS1 is suppressed by CO-releasing molecules [127,128].

**Figure 2:**
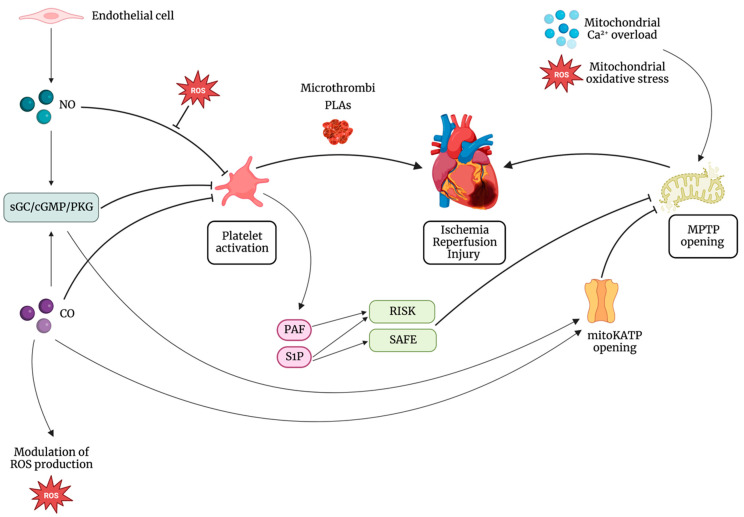
Mitochondrial permeability transition pore (MPTP) opening is the main end effector in ischemia reperfusion injury (IRI). On one side, platelets contribute to IRI mainly by microthrombi formation and platelet-leukocyte aggregates (PLAs). On the other side, molecules of platelet origin, such as platelet activating factor phosphoglyceride (PAF) and sphingosine-1-phosphate (S1P), lead to the activation of cardioprotective pathways, such as the reperfusion injury salvage kinase (RISK) pathway and the survivor activating factor enhancement (SAFE) pathway, all targeting inhibition of MPTP opening. Besides, the interaction between platelets and gasotransmitters has a central role in cardioprotection. Nitric oxide (NO) activates the soluble guanylate cyclase (sGC)/cyclic guanosine monophosphate (cGMP)/cGMP-dependent protein kinase (PKG) pathway, leading to the inhibition of platelet adhesion, activation, and aggregation. NO can inhibit platelet response also by cGMP-independent mechanisms. NO inhibitory effect on platelets is reduced in the presence of increased levels of reactive oxygen species (ROS). Also, PKG leads to the opening of the mitochondrial ATP-dependent K+ channel (mitoKATP) and subsequent inhibition of MPTP. Carbon monoxide (CO) exerts its cardioprotective action triggering the opening of mitoKATP with consequent inhibition of the opening of MPTP and modulating mitochondrial ROS production. Furthermore, it can inhibit platelet activation both by cGMP-dependent and cGMP-independent mechanisms.

The effect of CO on platelets may be also due to its effect on the cytochrome P450 enzymes with subsequent prevention of generation of arachidonic acid, a powerful proaggregating agent [129,130]. Figure 2 summarizes platelet–gases interactions in the cardioprotective scenario.

## 6. Conclusions

After years of intensive basic research, in vivo studies and ongoing clinical trials have provided evidence of the potential clinical relevance of the application of exogenous gasotransmitters and modulation of their endogenous production. However, large-scale clinical application is finding it difficult to be implemented, although the importance of gasotransmitter interaction has been suggested as a potential therapeutic strategy.

Gasotransmitters are among the molecules that have been shown to play a central role in triggering ischemic preconditioning [94,131,132] and mediating the effects of postconditioning [133,134,135]. Complex signaling pathways act synergistically in providing cardioprotection, and gasotransmitters are no exception. Several substrates, for example, undergo post-transcriptional modulation through nitrosylation of specific residues, and complex interactions between gasses have been demonstrated in several experimental models. It seems clear that most cardioprotective signaling pathways share an involvement of mitochondria, and several mitochondrial components have been shown to be selectively targeted by gases [136,137]. We have seen that two gasotransmitters, namely NO and CO, affect both platelets and cardioprotective pathways (Figure 2). Other gases, such as ROS, RNS, and H_2_S, are important in this field. Future studies may investigate whether the role of gases on platelets is necessary for cardioprotection.

## Figures and Tables

**Table 1:** The main stimuli and pathways involved in nitric oxide (NO) and carbon monoxide (CO)-induced effects on platelets. Abbreviations: eNOS endothelial nitric oxide synthase; iNOS inducible nitric oxide synthase; HO-1 heme oxygenase 1; cGMP cyclic guanosine monophosphate; HNO Nitroxyl; PKG protein kinase cGMP-dependent; IL-1β interleukin-1 β; PKC protein kinase C; ONOO^−^ peroxynitrite.

Gas	Stimuli	Production	Main Pathway	Effect(s)
NO	Shear stress, VEGF, insulin	Endothelial cells (eNOS)	NO/cGMP/PKG	Vasodilation
	[Ca]i increase, interaction protein (HSP70, HSP90, caveolin), insulin, β2 stimulation, acetylsalicylic acid, adenosine, and forskolin	Platelets (eNOS)	NO/cGMP/PKG	Reduction of adhesion, activation, and aggregation
	Inflammation	Platelets iNOS	NO/cGMP/PKG	Increased production of NO correlates with IL-1β
	Conditioning ischemia	Cardiac cells (eNOS or iNOS)	NO/cGMP/PKG S-nitrosylation	Cardioprotection
HNO	Conditioning ischemia	Cardiac cells (eNOS?)	PKCε translocation to the mitochondria	Cardioprotection
ONOO^−^	Metabolic diseases	NO + O_2_^−^	nitration carbonylation and peroxidation	Alteration of haemostatic functions
CO	Hemin and sodium arsenite	Platelet HO-1	cGMP/PKG	Reduction of aggregation and release of ADP and 5-HT
	Conditioning ischemia	Cardiac cells HO-1	Opening of KATP channel and closure of the MPTP.	Cardioprotection

## Data Availability

Not applicable.

## Abbreviations

ADP	adenosine diphosphate
ANP	atrial NP
ATP	adenosine triphosphate
BNP	brain NP
CNP	C-type NP
CO	carbon monoxide
CORM	CO donors
eNOS	endothelial NOS
HNO	nitroxyl
HO-1	heme oxygenase 1
iNOS	inducible NOS
IP	ischemic preconditioning
IR	ischemia reperfusion
IRI	ischemia reperfusion injury
JAK	Janus kinase
KATP	ATP-sensing potassium channels
LPS	lipopolysaccharide
mitoKATP	mitochondrial KATP channel
MOM	mitochondrial outer membrane
MPTP	mitochondrial permeability transition pore
NAC	N-acetyl-L-cysteine
NETs	neutrophil extracellular traps
NO	nitric oxide
NOS	nitric oxide synthase
NPs	natriuretic peptides
O_2_^−^	superoxide anion
ONOO^−^	peroxynitrite
P2Y12	purinergic Y-type 12
PAF	platelet activating factor phosphoglyceride
pGC	particulate GC
PKC	protein kinase C
PKG	cGMP-dependent protein kinase
PMN	polymorphonuclear
PMVs	plasma membrane-derived vesicles
PostC	Postconditioning
RIC	remote ischemic conditioning
RIPC	remote ischemic preconditioning
RiPerC	remote ischemic perconditioning
RiPostC	remote ischemic postconditioning
RISK	reperfusion injury salvage kinase
RNS	reactive nitrogen species
ROS	reactive oxygen species
S1P	sphingosine-1-phosphate
SAFE	enhancement of survival activating factor
sGC	soluble guanylate cyclase
SOD	superoxide dismutase
STAT3	signal transducer and activator of transcription 3
TXA_2_	thromboxane A_2_
VEGF	vascular endothelial growth factor
ZnPP-IX	zinc protoporphyrin IX
